# Facial mask for prevention of allergic rhinitis symptoms

**DOI:** 10.3389/falgy.2023.1265394

**Published:** 2023-12-06

**Authors:** Oğuzhan Oğuz, Felicia Manole, Nuray Bayar Muluk, Cemal Cingi

**Affiliations:** ^1^ Department of Audiology, Health Services Vocational School, Istanbul Nişantaşı University, Istanbul, Türkiye; ^2^ ENT Clinics, Dr. Oğuzhan Oğuz Wellnose Clinic, Istanbul, Türkiye; ^3^ Department of ENT, Faculty of Medicine, University of Oradea, Oradea, Romania; ^4^ Department of Otorhinolaryngology, Faculty of Medicine, Kırıkkale University, Kırıkkale, Türkiye; ^5^ Department of Otorhinolaryngology, Faculty of Medicine, Eskisehir Osmangazi University, Eskisehir, Türkiye

**Keywords:** face mask, allergic rhinitis, symptoms, aeroallergens, prevention

## Abstract

**Objectives:**

We reviewed the role of facial masks in preventing allergic rhinitis (AR) symptoms.

**Methods:**

The literature survey was performed in PubMed, EBSCO, UpToDate, and Proquest Central databases of Kırıkkale University and Google and Google Scholar databases.

**Results:**

Aeroallergens are microscopic airborne particles that trigger AR symptoms. In sensitive people, the type 1 hypersensitivity reaction against these allergens occurs when these microparticles enter the nasal mucosa via inhalation. Pollens, molds, dust mites, and animal dander are only some of the allergens suspected of contributing to AR symptoms. The treatment guidelines for AR extensively encompass allergy avoidance and environmental management as the first-line treatment. It is recommended that those who experience seasonal symptoms try to avoid their triggers whenever possible. While medical masks filter out particles larger than 3 μm, FFP2 masks are effective against particles as small as 0.004 μm. Since both mask types are effective in filtering pollen larger than 5 μm in size, they can be used to prevent pollen exposure. The “antiviral protection” provided by medical and FFP2 masks to hospital employees is roughly equivalent. Thus, both should be effective against direct local (eye) or indirect inhaled (nose, bronchial) pollen exposure. For the masks to do their job, they need to fit correctly.

**Conclusion:**

Face mask affects AR patients' quality of life and reduces AR symptoms' severity.

## 1. Introduction

Symptoms of the atopic disorder known as allergic rhinitis (AR) include a stuffy nose, clear rhinorrhea, sneezing, postnasal drip, and pruritis of the nose. Significant morbidity lost productivity, and healthcare expenses are connected with this condition, which affects one in six people. Previously, AR was only considered a nasal airway illness. However, advancing the unified airway hypothesis has defined AR as a component of a systemic allergic reaction, with related disorders sharing an underlying systemic pathology, such as asthma and atopic dermatitis (1). AR can be classed as seasonal (intermittent) or perennial (chronic), with about 20% of cases falling into the first category, 40% in the latter, and 40% exhibiting characteristics of both types. Patients with AR may additionally experience “non-productive cough, Eustachian tube dysfunction, chronic sinusitis, allergic conjunctivitis, and nasal symptoms” (2). Once AR has been diagnosed, multiple treatment options are available, the first of which is intra-nasal glucocorticoids (1, 3).

In this paper, we reviewed the role of facial masks in preventing allergic rhinitis (AR) symptoms.

## 2. Methods

The literature survey was performed in PubMed, EBSCO, UpToDate, Proquest Central databases of Kırıkkale University, and Google and Google Scholar databases. The keywords for the literature search are “face mask”, “facial mask”, “allergic rhinitis”, “symptoms”, “aeroallergens”, “FFP1 masks”, “FFP2 masks”, and “FFP3 masks”.

## 3. Allergic rhinitis

Despite its great frequency, allergic rhinitis (AR) is frequently misdiagnosed since it is a diverse illness with overlapping symptoms. Sneezing, itching, nasal congestion, and rhinorrhea are all possible symptoms. Pollens, molds, dust mites, and animal dander are only some of the allergens suspected of contributing to AR symptoms. Because seasonal allergic rhinitis (SAR) symptoms appear and disappear in predictable cycles in response to pollen exposure, the condition can be diagnosed with relative ease. There is considerable overlap between sinusitis, respiratory infections, and vasomotor rhinitis, making it more challenging to diagnose persistent AR than seasonal. Once pollen season is gone, SAR might cause an increased reaction to other allergens, such as cigarette smoke. According to the medical community, persistent AR manifests itself for about 9 months out of the year. Between 20% and 40% of people in the United States suffer from AR, and the prevalence is rising; 20% of cases are classified as SAR, 40% as perennial rhinitis, and 40% as mixed (2). The incidence of allergic rhinitis usually rises with age until early adulthood. Ongoing research is focused on exploring the impact of geographical factors on the epidemiology of allergic rhinitis, including the role of climate change (2).

Atopy runs in families, as does being male, having allergen-specific IgE, having a serum IgE level above 100 IU/mL before age 6, and being middle- or upper-class (5). According to studies, children who are introduced to solid meals or formula at a young age and/or who are exposed to secondhand smoke in large amounts during their first year of life are at a higher risk of developing AR (2). The health and well-being of individuals are significantly affected by air pollution and climate change, playing a role in the initiation and exacerbation of conditions such as allergic rhinitis and asthma, along with other chronic respiratory ailments (6). Various elements have been identified that may act as a buffer against the onset of AR. Despite the uncertainty about whether or not nursing contributes to the onset of AR, it is still strongly encouraged because of its many benefits and the lack of adverse side effects. A meta-analysis of 8 research found a 40% decreased risk in people who had lived on a farm during the first year of their lives, sparking a growing interest in the “farm effect” on the development of allergies (7).

It is recommended that those who experience seasonal symptoms try to avoid their triggers whenever possible. Dust mites, animal dander, and upholstery can all be avoided with precautions, but doing so may necessitate significant lifestyle adjustments that the patient is unwilling to do. If removing a pet from the home is not an option, keeping it in a separate room may help reduce exposure to pet dander. Even after the cat is gone, the dander it left behind could linger for up to 20 weeks. A vacuum cleaner with high-efficiency particulate air (HEPA) filters and an allergen-proof mattress cover may help alleviate symptoms (5).

## 4. Facial masks

Medical or surgical face masks are considered medical devices (MDs) (8).

Respiratory protective gear, such as filtering facepiece (FFP1, FFP2, and FFP3) masks, which are classified as PPE (8).

Fabric/cloth masks sold in stores for the general public are not personal protective equipment (PPE) under Regulation EU/2017/745 (9) nor are they medical devices (MDs) under Regulation EU/2016/425 (10).

Fabric/cloth masks manufactured at home by anyone for their usage are called “Confection artisanal” in French (11) or “Do it Yourself” (DIY) masks in English. The World Health Organization (12) classifies masks made from polypropylene and other woven and non-woven fabrics (NWFs) as “non-medical” or “fabric” masks. WHO has reported (12):

Non-medical fabric masks can be constructed from an infinite variety of fabrics and materials in a wide range of layering sequences and shapes; however, only some of these combinations have been systematically evaluated, and there is no standard design, material, or shape (12).

The French Standardization Association (AFNOR Group) has developed a standard for non-medical masks “to define minimum performance in terms of filtration (minimum 70% solid particle filtration or droplet filtration) and breathability (maximum pressure difference of 0.6 mbar/cm^2^ or max inhalation resistance of 2.4 mbar and max exhalation resistance of 3 mbar)” (13).

While medical masks filter out particles larger than 3 μm (14), FFP2 masks are effective against particles as small as 0.004 μm. Since both mask types are effective in filtering pollen larger than 5 μm in size (15, 16), they can be used to prevent pollen exposure. The “antiviral protection” provided by medical and FFP2 masks to hospital employees is roughly equivalent (17). Thus, both should be effective against direct local (eye) or indirect inhaled (nose, bronchial) pollen exposure.

For the masks to do their job, they need to fit correctly. Researchers have found that good mask fitting may be practiced in relatively brief training regimens (18). Therefore, it is advised that all healthcare personnel undergo fit tests, including those using bitter liquids (19).

## 5. AR and facial mask

Without a face mask, the respiratory mucosa would be exposed to the coronavirus particles in Vero cells and droplets, triggering immunological and inflammatory responses. Symptoms of Allergic Rhinitis (AR) or a viral infection are triggered by these (20).

Aeroallergens are microscopic airborne particles that trigger AR symptoms. In sensitive people, the type 1 hypersensitivity reaction against these allergens occurs when these microparticles enter the nasal mucosa via inhalation (21). Treatment choices encompass strategies such as minimizing exposure to airborne allergens and environmental controls, as well as utilizing single or combined pharmacotherapy options (steroids, oral or intranasal antihistaminics) and allergen immunotherapy (22).

The mucosa of the respiratory system should not come into touch with allergens, and environmental controls work toward that end. By minimizing the patient's exposure to allergens, preventative measures can boost treatment success and enhance the quality of life (23). The treatment approach for allergic rhinitis often involves discussions about allergen avoidance and the implementation of environmental controls (ECs), in addition to the use of pharmacological interventions and allergen immunotherapy (AIT) (7). Furthermore, avoiding exposure to outdoor allergens is considerably more complex than interior ones. As the leading trigger of allergic rhinitis, pollens are ubiquitous in outdoor environments (24). Pollen, the minute male reproductive unit of plants, is highly allergic due to the presence of several allergenic proteins. Using a face mask during peak pollen times is a recommended technique for avoiding pollen in AR patients who are sensitive to it (25).

Patients utilizing nasal filters showed significant improvement in various nasal symptoms in a placebo-controlled study of 24 patients exposed repeatedly in an environmental exposure unit, as described by Kenney et al. (26). However, there was no statistically significant improvement in the overall allergy score (26). A 15-item questionnaire was designed following the study's aims by a group of experts in allergic rhinitis by Mengi et al. (27). Patients' nasal and ocular symptoms improved after mask use (*p *= 0.001). Sneezing (*p* = 0.029) and nasal discharge (*p* = 0.039) decreased the most compared to baseline allergy symptom levels. They found that people with pollen allergies experienced fewer nasal and ocular allergic rhinitis symptoms when they used face masks (27).

Using a surgical face mask and an N95, Dror et al. (28) evaluated the nurses who had reported AR. The study revealed an improvement in mild AR symptoms while using a surgical face mask but no change in mild AR symptoms when using an FFP2 face mask (28). It is safe to presume that allergens such as pollen, mold spores, animal fur, and house dust mites are at deficient concentrations within a hospital (17). Participants with allergic rhino-conjunctivitis symptoms were exposed to outdoor allergens while wearing either a medical face mask or an FFP2 face mask in a separate trial by Bergmann et al. (11) during the COVID-19 pandemic. They felt better overall, and their symptoms lessened than before the pandemic and when they were not wearing masks (17).

In their study (20), Esmaeilzadeh et al. looked at how wearing a face mask affected the severity of AR symptoms in instances of AR during the Corona Virus Disease 2019 (COVID-19) pandemic by utilizing “the Sino-Nasal Outcome Test (SNOT-22)”. Before and after the pandemic, AR symptoms were compared using “the SNOT-22 survey”. They determined that wearing a face mask during the COVID-19 pandemic positively affected AR patients' quality of life and reduced the intensity of AR symptoms. This harshness is amplified by smoking. There was no correlation between the intensity of AR symptoms and changes in quality of life during the COVID-19 pandemic and age, gender, pet ownership, underlying illnesses, or prior infection with COVID-19.

## 6. Conclusion

Millions of individuals globally experience a diminished quality of life due to allergic rhinitis (29). Aeroallergens are microscopic airborne particles that trigger AR symptoms. In sensitive people, the type 1 hypersensitivity reaction to these allergens occurs when these microparticles are inhaled and land on the nasal mucosa. Wearing a face mask can significantly impact the quality of life and reduce the severity of AR symptoms.
